# Association between alcohol sales and facial fracture rates: an ecological analysis

**DOI:** 10.1093/alcalc/agaf006

**Published:** 2025-02-20

**Authors:** Annamari Arpalahti, Johanna Snäll, Jussi Kanervo, Aleksi Haapanen, Anna Liisa Suominen, Johanna Uittamo

**Affiliations:** Department of Oral and Maxillofacial Diseases, University of Helsinki and Helsinki University Hospital, FI-00290, Helsinki, Finland; Department of Oral and Maxillofacial Diseases, University of Helsinki and Helsinki University Hospital, FI-00290, Helsinki, Finland; Department of Oral and Maxillofacial Diseases, University of Helsinki and Helsinki University Hospital, FI-00290, Helsinki, Finland; Department of Oral and Maxillofacial Diseases, University of Helsinki and Helsinki University Hospital, FI-00290, Helsinki, Finland; Institute of Dentistry, University of Eastern Finland, FI-70211 Kuopio, Finland; Oral and Maxillofacial Teaching Unit, Kuopio University Hospital, FI-70211, Kuopio, Finland; Department of Public Health, Finnish Institute for Health and Welfare (THL), FI-00300, Helsinki, Finland; Department of Oral and Maxillofacial Diseases, University of Helsinki and Helsinki University Hospital, FI-00290, Helsinki, Finland

**Keywords:** facial fractures, interpersonal violence, alcohol sales

## Abstract

**Aims:**

This study aimed to evaluate national alcohol sales and their association with the number of maxillofacial fractures in Southern Finland.

**Methods:**

Patient data of all facial fracture patients admitted to tertiary trauma centers (Helsinki University Hospital, Helsinki, Finland) from January 2014 to October 2020 were reviewed retrospectively. Information on alcohol sales in Finland was obtained from the Finnish Institute for Health and Welfare.

**Results:**

The annual number of facial fractures increased, as did the number of facial fractures caused by interpersonal violence. Unexpectedly, we found a mostly inverse association between alcohol sales and facial fractures, although three months were associated positively: April, June, and November.

**Conclusion:**

We conclude that although the significance of alcohol use in the etymology of facial fractures has been unmistakably proven neither population-level alcohol use nor interpersonal violence as an injury mechanism explains the increase in facial fractures. However, there are some associations between the seasonality of alcohol consumption and facial fractures, suggesting the same predisposing factors in both. Further, certain groups of users, exceeding a threshold of alcohol use, appear to be responsible for the traumatic presentations in emergency units. Elucidating the associations between alcohol use and facial fractures requires an assessment of patient-specific factors, rather than population-level alcohol use, for a detailed understanding and justification of alcohol policy.

## Teaser

This study evaluated the association between alcohol sales and facial fractures. This turned out to be generally inverse, although the proportion of assault-related fractures remained stable. However, some seasonality between alcohol sales and facial fractures was appreciated, suggesting similar predisposing factors. Polarization in alcohol use might explain these results.

## Introduction

The burden of alcohol-related diseases is borne by over 200 different diagnoses (Im et al. 2023). There is no safe level of alcohol consumption—even light drinking contributes to health risks (Collaborators 2018).

Alcohol also plays a significant role in various traumas, including interpersonal violence (IPV), traffic accidents, and falls (Hayman and Crandall 2009). A review by Kool et al. indicates that alcohol consumption increases the risk of falls by 3- to 4-fold among young and middle-aged patients (Kool et al. 2009). Additionally, Kalsi et al. reported a correlation between alcohol use and fatal traffic accidents in Finland (Kalsi et al. 2018). Following a reduction in alcohol taxes in 2004, consumption increased by 12.4%, leading to a 38% rise in alcohol-related motor vehicle accidents (MVAs) that resulted in death (Kalsi et al. 2018).

The facial skeleton consists of several bones that form the structure of the face. These bones provide support for facial muscles and protect vital structures such as the eyes, nose, and mouth. The bony injuries of the neurocranium are the area of expertise for the neurosurgeons and the facial areas are for the maxillofacial surgeons, respectively. Regarding all facial fractures, up to 55% of patients have been under the influence of alcohol when the injury occurred (Hirvikangas et al. 2020). In particular, alcohol use is associated with IPV-related facial fractures (O'meara et al. 2012, Arpalahti et al. 2023).

A tertiary trauma center can provide definitive care for all injured patients. In Finland, five tertiary trauma centers operate as part of the country’s university hospitals. Facial fractures are treated mainly in these tertiary trauma centers. Primary and secondary trauma centers such as health stations, healthcare centers, and intermediate-level care hospitals participate in the process by treating minor injuries and primarily diagnosing and referring facial fracture patients to tertiary centers.

Alcohol policy in Finland underwent significant changes in 2018. Before these major reforms, Finland had some of the strictest alcohol policies in Europe, aimed at reducing alcohol-related harms. The state-owned alcohol retail seller Alko had exclusive rights to sell alcoholic beverages with more than 4.7% alcohol by volume (ABV). In addition, there were strict limitations on alcohol advertising, aimed particularly at protecting minors. In 2018, alcohol law reform increased the availability in grocery stores of alcohol with up to 5.5% ABV. Restaurants and bars were granted more flexibility in their serving hours. Before the new legislation, alcohol service ended at 1:30–3:00 a.m., but the new law enabled service until 4:00 a.m.

The aim of this study was to evaluate alcohol sales in Finland and their association with the increasing number of facial fractures.

## Materials and methods

### Study design and data collection

Data on all facial fracture patients admitted to tertiary trauma centers (Helsinki University Hospital, Helsinki, Finland) from January 2014 to October 2020 were assessed. Patients were identified according to facial fracture diagnosis codes (ICD-10: S02* craniofacial fractures) from the electronic patient data by reviewing medical records. All patients with clinically diagnosed and radiologically confirmed craniofacial fractures were included in the study. Excluded were patients with solely cranial fractures. The numbers of facial fractures (monthly and yearly) were recorded, and their etiologies were classified as assault, fall, traffic accident, or other.

Data from the same period on alcohol sales in Finland were obtained from the Finnish Institute for Health and Welfare (THL) and comprised monthly sales in three categories: alcohol dispersing, alcohol retail, and total alcohol sales combining the preceding two sales numbers.

### Statistical analysis

Pearson correlations were calculated separately between total alcohol sales and numbers of facial fractures in each month in the combined data between 2014 and 2020. Linear regressions were further used to analyze statistical significance between total alcohol sales and number of facial fractures in each month.

The relationship between alcohol sales and the number of facial fractures was observed and visualized through line charts, histograms, and their combinations.

## Results

**Figure 1:**
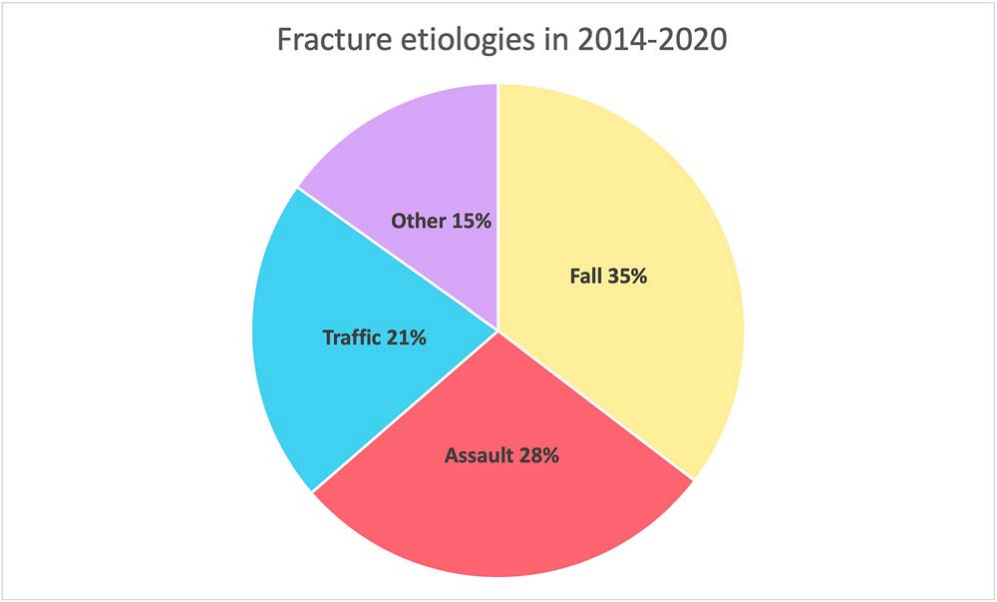
The three most common etiological factors in facial fractures were falls, IPV, and traffic-related accidents, which included accidents with motor vehicles, bicycles, and electric scooters.

Overall, data of 3731 facial fracture patients were compared with alcohol sales. Figure 1 visualizes the main etiologies behind facial fractures; falls (36%) and assaults (28%) were the main injury mechanisms, and the remainders were caused by traffic-related injuries (21%) or other etiologies (15%).

**Figure 2:**
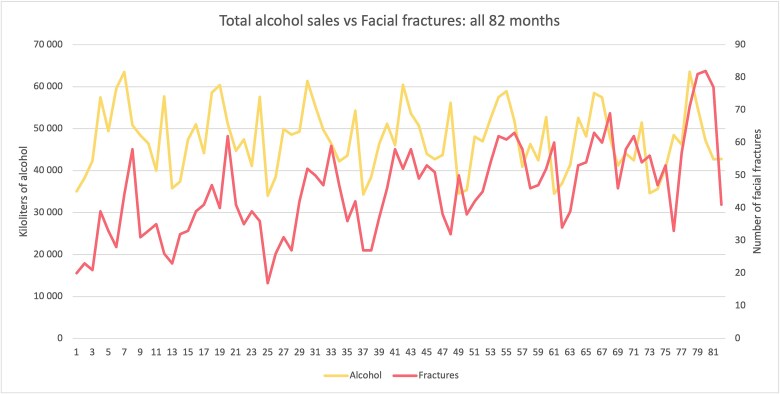
Figure 2 presents the values for total alcohol sales and number of facial fractures in Finland for all 82 months of the study period

**Figure 3:**
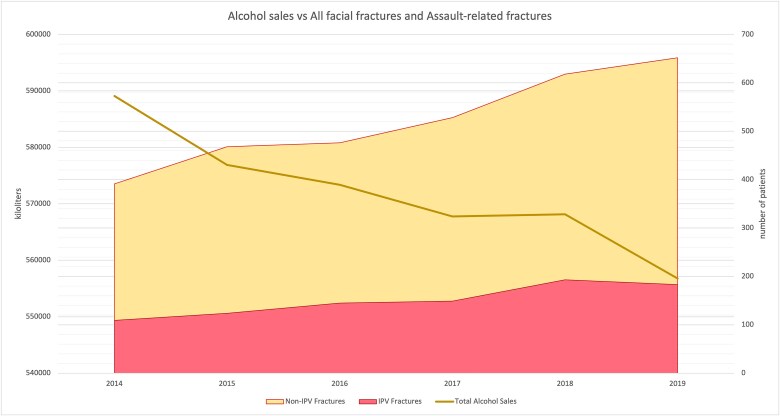
Figure 3 visualizes the relation between total alcohol sales in Finland (dark yellow line) and number of fractures (highest red line), differentiating non-IPV-related fractures (yellow AUC) and IPV-related fractures (red AUC). A moderate decline in alcohol sales occurred, while a relatively steady incline described the trend for facial fractures. Despite the decline in alcohol sales, the proportion of assault-related fractures remained relatively stable at slightly under one-third of all facial fractures

The numbers for all 82 months of the study period for alcohol sales and facial fractures are presented in Fig. 2. The annual numbers are presented in Fig. 3 to more succinctly visualize the trends. Through 2014 to 2020, a steady decline in alcohol sales occurred. By contrast, a steady incline occurred in the incidence of facial fractures. Assault-related fractures, visualized as the red area under the curve (AUC), climbed steadily, maintaining a role in fracture etiology despite declining alcohol sales. Alcohol sales included retail and dispersing, accounting for 85% and 15% of all sales, respectively.

**Figure 4:**
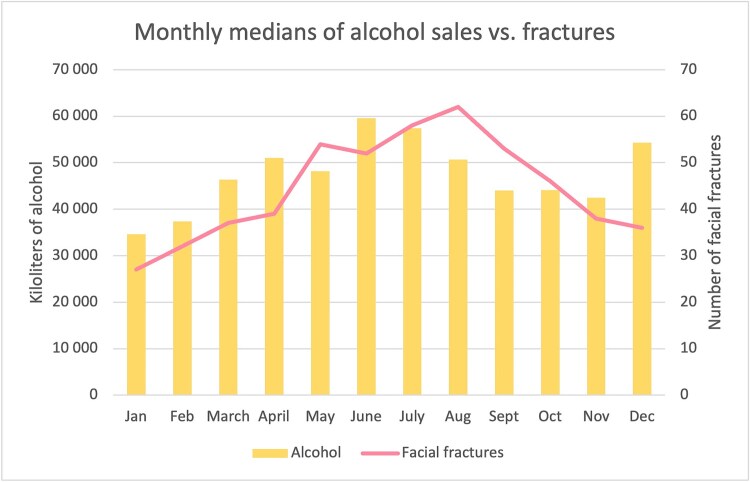
The relationship between all alcohol sales and number of facial fractures is presented using median values for each month of the year. In general, alcohol consumption increased markedly during the summer months, declining toward winter. Surprisingly, the correlation between alcohol sales and facial fractures is generally seen as inverse; as sales increase, the number of facial fractures decreases. However, there are three months in which the correlation is positive, albeit weak (April c = .243, June c = .257) or very weak (November c = .139). The strongest negative correlations were observed in December (c = −.940), February (c = −.906), and July (c = −.596). Using linear regression, inverse associations were statistically significant for February and December (*P* < .001) as well as for July (*P* = .049).

The relation between monthly alcohol sales and diagnosed facial fractures is illustrated in Fig. 4 using median values for each month of the years 2014 and 2020. In general, summer was the most common time for fractures. Trauma peaks correspond to vacation periods such as Easter and May Day with a similar trend in alcohol sales.

An inverse correlation was generally seen between monthly alcohol sales and facial fractures; as sales increased, the number of facial fractures decreased. However, there are three months in which the correlation was positive, albeit weak (April c = .243, June c = .257) or very weak (November c = .139). The strongest negative correlations were observed in December (c = −.940), February (c = −.906), and July (c = −.596). Using linear regression, inverse associations were statistically significant for February and December (*P* < .001) as well as for July (*P* = .049).

## Discussion

This study aimed to evaluate alcohol sales in Finland and their correlation with the increasing number of facial fractures.

Our figure for alcohol sales revealed a subtle declining trend, a development also shown for alcohol consumption in Scandinavia in a status report by the World Health Organization (WHO), describing changes in alcohol consumption during 2010–2016 (World Health Organization 2019). Across the European Union (EU), this decline was not statistically significant (World Health Organization 2019). A significant downward development has been noted in Russia (Radaev and Roshchina 2021). By contrast, alcohol consumption has increased in the United States (US) (Dawson et al. 2015, Kerr et al. 2022), India (Rastogi et al. 2022), and the Mediterranean, although the latter to a lesser degree (Rostam-Abadi et al. 2024).

Despite Finland having a decrease in alcohol consumption, it has the worst mortality rates related to alcohol use among the Scandinavian countries (World Health Organization 2019). WHO reported a decline in those currently drinking and those binge drinking as well as a slight rise in the amount of alcohol consumed individually in the EU (World Health Organization 2019). Taken together, Finnish alcohol consumption appears to be polarized to certain user groups, while in the general population the use of alcohol has decreased. No single consumption trend predominates globally. In the US, increases in both daily use and alcohol use disorders after the pandemic have been identified (Kerr et al. 2022). By contrast, while alcohol consumption overall has increased in India, the amount of daily drinking has diminished (Rastogi et al. 2022) – possibly pointing to a polarizing development where those who already drink heavily consume increasingly more alcohol, a trend suggested at least in the US (Hingson et al. 2017, Kerr et al. 2022). In Russia, the findings of increased heavier drinking despite declining general use are suggested to stem from a collectivity hypothesis (Radaev and Roshchina 2021). In any event, the rising use of alcohol overall burdens the healthcare system and warrants attention.

When questioned on the amount of alcohol consumed when patients presented to the emergency room with facial fractures, they were more likely to admit to having consumed larger amounts of alcohol (Hirvikangas et al. 2020). Taken together, it seems that alcohol accounts for fracture incidence only after a certain threshold, after which aggression and impulsivity increase significantly in individuals. Across the literature, no single prevailing seasonal trend for facial fractures dominates globally: in South Australia, it is autumn (Diab et al. 2022), in the Netherlands spring (Van Hout et al. 2013), and in Texas summer (Morris et al. 2015). In women, whose facial fractures involve inebriation less often than in men, summertime is the season with the least fractures (Diab and Moore 2023). For comparison, for fractures of the extremities in adults, there seems to be a predilection for the winter months (Wareham et al. 2003, Hoff et al. 2016, Kruse et al. 2022), while for clavicular fractures seasonal differences are not deemed significant (Postacchini et al. 2002).

In our results, some monthly peaks correlated with alcohol consumption and facial fractures. Strong negative correlations were found for December, February, and July. These are months, where people often may travel outside of their home counties—such holidays include winter break in February, summer vacation in July, and Christmas in December. These holidays usually involve time spent with family outside of the Helsinki metropolitan area and not necessarily much alcohol but may involve other reasons for fractures such as falls. They may also cause the treatment itself to occur at a different University Hospital. Trauma peaks seem to occur over the holidays, which also have the heaviest alcohol consumption. Specifically, positive correlation was found for April, June, and November. These months have many public holidays involving the use of alcohol and spending time in public with others, such as Easter and pre-Christmas parties.

While these speculations are justifiable, it is also important to take into consideration that the relationship between alcohol and risky behavior is not always straightforward—the highly educated have been suggested to consume alcohol above the risk limit but the negative consequences do not tend to accumulate because there are usually less coinciding life difficulties to make the situation worse. Additionally, this study consists of a large study material, where large-scale trends can be seen: in the case of alcohol and facial fractures, the general trend seems to be a negative correlation between the two.

It is noteworthy that the alcohol data included only alcohol sales in Finland, excluding imports from other countries. Before the pandemic, import was estimated to constitute 15% of total sales, and in 2022 the estimate was 9%, the vast majority (~70%) comprising beer and cider (Finnish Institute for Health and Welfare 2024), suggesting a similar declining trend in alcohol consumption in total. However, at the same time, the use of other substances may have an influence on injury occurrence. The last two months (November and December) of 2020 were excluded from the statistics due to changes in the electronic patient record system and the restrictive measures for Covid-19 coinciding with the same period. We concluded that the information from that period was not sufficiently reliable to be included in the data. This exclusion might make the results of 2020 deviate slightly from the trends of earlier years.

Although in this study we did not show an association between alcohol sales and incidence of IPV-related facial fractures, individual-level effects may be considerable. The relationship between alcohol and IPV-related facial injury is widely known—IPV as the cause is highlighted when alcohol is involved (Goulart et al. 2015, Lee and Qiu 2017, Hirvikangas et al. 2020)—and the injuries are more pronounced the more alcohol consumed (Hingson et al. 2017). With female patients, alcohol may increase the incidence of IPV-related fractures occurring in public (Gerber et al. 2009). Alcohol has also been shown to increase the number of fractures on the facial region (Lee and Qiu 2017) and the severity of the injuries (Lee and Qiu 2017, Othman et al. 2020). Therefore, it is essential to direct our resources not solely to the general consumption of alcohol but also to heavy drinking to combat the synergy between alcohol and IPV.

In conclusion, the number of facial fractures has increased in recent years, but alcohol consumption seems to have decreased in the population over the same period. Neither population-level alcohol use nor IPV as an injury mechanism explain the increase in facial fractures. However, there are some associations between the seasonality of alcohol consumption and facial fractures, suggesting the same predisposing factors in both.

## Data Availability

Due to ethical reasons, the full data on facial fracture patients is not published online to protect patient confidentiality. Alcohol sales statistics are available in Valvira (The National Supervisory Authority for Welfare and Health, working under THL) website (https://valvira.fi/alkoholi/tilastot), obtained in the summer of 2023. The statistics on the page include 5 years of the most recent data and are in Finnish – older information is stored in Alcohol trade register Allu (https://valvira.fi/en/alcohol/alcohol-trade-register). The statistics and graphs derived from these statistics are attached to this paper.
